# On Fracture of the Sternum

**Published:** 1807-11

**Authors:** T. Bishopp

**Affiliations:** Member of the Royal College of Surgeons


### *On Fracture of the Sternum; by* Mr. T. Bishopp, *Member of the Royal College of Surgeons.*

In the Medical and Physical Journal, No. 103, is a valuable paper on Fractured Sternum, by the celebrated M. Sabatier. By this paper it appears, that he employed in his cases only a *loose* bandage, with a view simply to retain on the part his medicinal applications. He did not try a strict bandage, not because, as it should seem, that he had before actually found it to be either useless or injurious, but because he imagined it must be useless. Now, with the great utility of a tight bandage around the ribs, when any of them are fractured, he was unquestionably sufficiently acquainted; but, that this would likewise be useful in certain cases of fractured sternum, whose circumstances are very similar, appears not to have occurred to him. Whether such compression may answer a good purpose in all circumstances of this fracture, I am very incompetent to say. Whenever spiculæ of bone can be supposed to have been driven inwards, any further depression would certainly endanger the internal mammary vessels, &c.; but the pressure produced on the sternum by a circular bandage is very little, often none at all. That it succeeded to my utmost satisfaction in a single instance, will appear from the following narrative.

George Popple, ætat. 36, a very robust man, a carrier, was knocked down by two ruffians, who after having beaten him most violently with a bludgeon on the breast, head, and arms, imagined they had killed him; yet, in order more completely to effect this purpose, one of them leaped upon the breast of the unhappy victim of their brutality whilst lying on his back. The poor man, still retaining his senses, now perceived a copious effusion of blood from his mouth and nostrils, and the heart and lungs to be pressed violently as it were up into his throat. In this condition the villains left him. After two or three hours he was brought to Leicester, two miles distant from the fatal spot, in almost a lifeless state. I found him, about eleven o'clock, P. M. very cold and faint, with a faultering, labouring pulse, breathing much oppressed, leaning forwards, and unable to speak. Upon undressing him, I discovered that the sternum was broken into three irregular portions; the os brachii of the one, and the radius and ulna of the other arm were also broken. I could not ascertain that any of the ribs had suffered in the same way.

way. After he had become sufficiently warm in bed, I bled him freely, and bound up the chest with a tight napkin; gave him an opiate, and directed that he should take nothing but common tea. The next morning he was extremely ill, respiration excessively laborious, cough incessant, with bloody expectoration; great vascular excitement, together with great pain and oppression in the chest, complaining much of a pricking pain under the sternum, and of stitches in his sides whenever he coughed; he could breathe only when nearly half erect in bed. I now bound the ribs very firmly with a broad inelastic girth, furnished with four straps and buckles, when he exclaimed, "he was in heaven." I bled him very freely again, twice or thrice, purged him severely every morning, and added to the strictest antiphlogistic plan a nightly opiate. He went on well till the morning of the fourth day, when a servant hastened to call me to his assistance; as, upon her raising him up to give him some food, he instantly screamed, fell backwards, and appeared to be gasping for his last breath. I found him black in the face, frothing at the mouth, the eyes staring, in the greatest imaginable agony. Staggered at this alarming appearance, I looked if the bandage had been loosened; which had happened; and upon tightening it, the problem was solved by the speedy cessation of the more distressing effects, and by his becoming tranquil and easy in less than ten minutes. The bandage had slipped down. To obviate the recurrence of such a disaster, shoulder-straps were attached to it. The case went on afterwards without a single difficulty. In about eight weeks he was able to ride on horseback. The fractured sternum had reunited perfectly with little or no irregularity; for it should have been before stated, that one portion was not depressed at all under another.

It is not, I apprehend, the coaptation of the bone that is of main consequence in these accidents; Nature will commonly adjust all this part of the business, and effect the reunion, whatever be the new relative position of the fragments. The object most deserving our immediate attention is the present performance of respiration in the most easy manner possible. The anterior extremities of the ribs are here no longer the fixed points they were before, consequently the muscles soon become so much fatigued, that they can act no longer, or they act with great difficulty; all our dependance therefore must be upon steadying the ribs, by applying to them a firm lateral support. It is, then, to fatigue and spasm of the intercostal and and other muscles concerned in respiration, and not to the mechanical irritation from the fractured bone, that I would generally attribute the greater part of the distress produced, ab initio, from this accident; accordingly, my patient felt as if his body were parting into pieces when he was unbound.

*Leicester, October* 1807.

